# *Salmonella* Enteritidis and *Salmonella* Typhimorium identification in poultry carcasses

**Published:** 2018-02

**Authors:** Asma Afshari, Ahmad Baratpour, Saeed Khanzade, Abdollah Jamshidi

**Affiliations:** 1 Department of Nutrition, School of Medicine, Mashhad University of Medical Sciences, Mashhad, Iran; 2 Department of Food Hygiene, School of Veterinary Medicine, Ferdowsi University of Mashhad, Mashhad, Iran

**Keywords:** *Salmonella* Enteritidis, *Salmonella* typhimurium, Poultry carcasses, Zoonotic diseases

## Abstract

**Background and Objectives:**

Salmonellosis caused by *Salmonella* spp. is one of the most important zoonotic diseases and transmits to human through raw food animal products including poultry meat. *Salmonella enterica* serovar Enteritidis and *Salmonella enterica* serovar Typhimurium are the most important strains that infect human. This study was conducted to evaluate the contamination rate of poultry carcasses with *S.* Enteritidis and *S.* Typhimurium using multiplex PCR assay.

**Materials and Methods:**

100 samples were selected during the summer and fall of 2010 by cluster sampling method from 10 broiler flocks, which were slaughtered in a poultry abattoir located in Mashhad suburb. After culturing the samples in enrichment and selective media and obtaining suspected colonies, DNA was extracted and *Salmonella* isolates were identified by multiplex-PCR. Three sets of primer pairs tagreting *invA* gene for *Salmonella* genus, *prot6* gene for entritidis serovar and *fliC* gene for Typhimurium serovar were used.

**Results:**

The contamination of poultry carcasses with *Salmonella* was 14% (14/100) which 43% (6/14) of them were identified as *S*. Enteritidis and 36% (5/14) identified as *S.* Typhimurium, respectively.

**Conclusion:**

Results of this study indicated that the risk of zoonotic diseases created by *S*. Enteritidis and *S.* Typhimurium is relatively high in poultry carcasses.

## INTRODUCTION

*Salmonella* is a Gram-negative, facultative anaerobic bacteria, belonging to the family of *Enterobacteriaceae* (1). *Salmonella* spp. is widely distributed in the environment, but the intestinal tract of animals is the main habitat of the bacteria (2). *Salmonella* contamination occurs through the consumption of contaminated foods like egg, milk and poultry meat (3). Twenty percent of world poultry products are contaminated with *Salmonella*, and they can persist for a long time in the animal and human environments and facilities through biofilm formation (4). In most of the salmonellosis outbreaks resulted from poultry products consumption, Enteritidis and Typhimurium serovars have been isolated (5). In Iran, poultries are reported to be the predominant reservoirs for *Salmonella enterica* and serovar Enteritidis was isolated in 51.4% (35/68) of the samples (6). *S. enterica*, serovar Enteritidis is implicated in 60% of salmonellosis in European people and is the world’s leading cause of salmonellosis (7). In the United States, *S.* Typhimurium is mostly associated with Salmonellosis (8).

Multidrug-resistant (MDR) due to *Salmonella* is known as a major public health problem around the world and there is an increased use of antibiotics in human and animal settings (9). MDR *Salmonella* may be transmitted to human throughout the production chain and so important risk factors have been identified during processing (10).

Polymerase Chain Reaction (PCR) is a valuable method for investigating food-borne outbreaks and pathogens identification (11). PCR provides fast results and a high degree of specificity. The incorporation of a routine PCR test in combination with traditional culture methods could be effective in providing a more accurate profile of the prevalence of this pathogen in broiler carcasses (12).

Because the main source of carcasses contamination with *Salmonella* are intestinal tract, skin and feathers of chickens, which may develop along the processing line (13), this study was conducted on carcasses of a slaughterhouse to evaluate the contamination rate of poultry carcasses with *Salmonella* spp., especially *S*. Enteritidis and *S*. Typhimurium in a poultry processing plant in Mashhad, Iran, using culture and multiplex PCR method by detecting *prot6E* and *fliC* genes.

## MATERIALS AND METHODS

### Sampling method.

Sampling was conducted in an abattoir located in suburb of Mashhad city, Iran. In this study, a total of 100 samples were collected from 10 broiler flocks by cluster sampling method. Samples were chosen after the chiller step in the processing line as followed: excess fluid from the carcass was aseptically drained and the carcass was transferred to a large sterile plastic bag. 400 mL buffered peptone water was poured into each bag containing carcass. Inside and outside of the bird carcasses were rinsed with a rocking motion for one minutes and the rinse fluid was transferred to a sterile container. Plastic bags containing rinsed fluid were immediately transported to the laboratory inside a portable ice-chest and bacterial analyses were started within 1–4 h (14).

### Isolation and identification of bacteria.

Rinse fluid was transferred into a 50 mL falcon tube and centrifuged at 4000 rpm for 10 minutes. Precipitates were suspended in 9 mL of lactose broth (Merck, Germany) and incubated at 37°C for 24 h. Amount of 1 mL of each re-enriched sample was transferred into 9 mL of selenite cystine broth (Bio-Rad) and incubated at 37°C for 24 h. Following incubation, a loopful of each culture was streaked onto brilliant green agar (Bio-Rad) which was incubated at 37°C for 24 h. Presumptive *Salmonella* colonies were confirmed biochemically using triple sugar iron (TSI), citrate, lysine decarboxylase, urease and indole tests (15).

### DNA extraction.

The bacterial colonies that were confirmed as *Salmonella* spp. by biochemical tests were cultured overnight on nutrient agar. Then, DNA extraction was performed using boiling method (16).

### Multiplex PCR.

PCR reaction was performed in a final volume of 25 μl containing 2.5 μl 10x PCR buffer (500 mM KCl, 200 mM Tris–HCl), 1.25 μl deoxynucleotide triphosphate (10 mM), 1.5 μl MgCl_2_(2 mM), 0.5 μl Taq DNA polymerase (Fermentas), 2 μl extracted DNA and 0.5 μl each primer. Amplification reaction and cycling condition were performed in a thermal cycler (Techne, UK) as follows: an initial incubation at 95°C for 5 min, followed by 35 cycles of denaturation at 94°C for 60 s, annealing at 56°C for 30 s, extension at 72°C for 30 s, and a final extension period for 10 min at 72°C (17). Amplified products were resolved in 1.2% agarose gel and photographed under UV light.

Primers of S139 and S141 which were specific for *invA* gene were used for detection of the genus *Salmonella* and primers of prot6e-6 and prot6e-5 which were specific for *prot6E* gene were used to identify *S*. Enteritidis and Fli15 and Tym primers which were specific for *fliC* gene were used to identify *S.* Typhimurium (Table 1). *S*. Enteritidis (ATCC-13076) and *S*. Typhimurium (ATCC: 14028) were used as positive controls and sterile distilled water as negative control.

**Table 1.** Sequence of oligonucleotides used as primers in the multiplex-PCR.

**primers**	**sequences (5′–3′)**	**Target gene**	**Product size (bp)**	**References**
*S139*-F	GTG AAA TTATCG CCA CGT TCG GGC AA	*InvA*	284	(18)
*S141*-R	TCA TCG CAC CGT CAA AGG AAC C			
*Fli15*-F	CGG TGT TGC CCA GGT TGGTAAT	*fliC*	559	(19)
*Tym*-R	ACT CTT GCT GGC GGT GCG ACTT			
*Prot6e*-5-F	ATA TGG TCG TTG CTGCTT CC	*Prot6e*	185	(20)
*Prot6e-6*-R	CATTGT CCA CCG TCA CTTTG			

## RESULTS

**Fig. 1.**
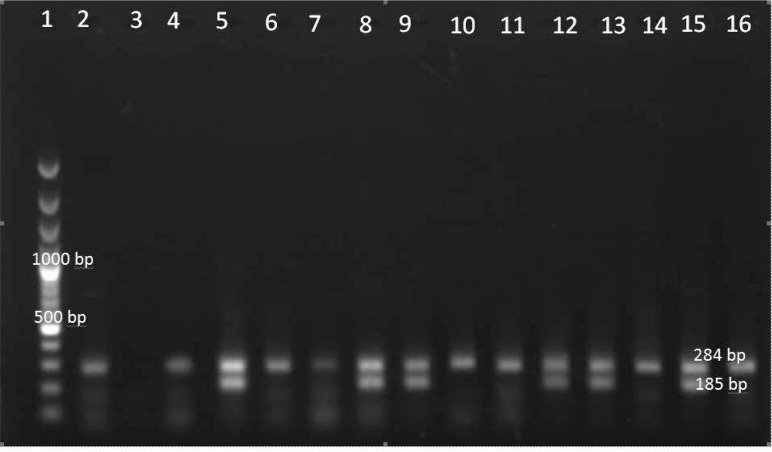
Multiplex-PCR assay using three sets of primers. The 284 bp amplified product from *invA* gene specific for *Salmonella* spp., and prot6e amplified a 185-bp for *S*. Entritidis. Lane 1: 100 bp DNA ladder; Lane 2: positive control for *S*. Entritidis; Lane 3: negative control; Lanes 4, 5, 6, 7, 8, 9, 10, 11, 12, 13, 14, 15, 16: positive samples for *Salmonella* spp. Lanes 5, 8, 9, 12, 13, 15: positive samples for *S*. entritidis.

**Fig. 2.**
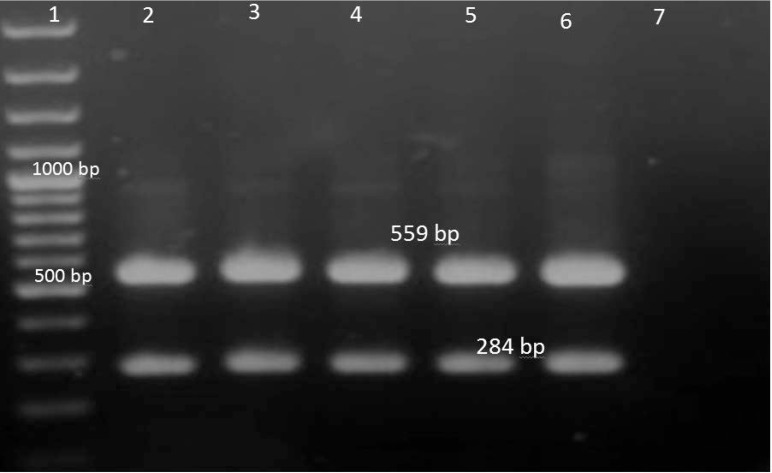
Multiplex-PCR assay using three sets of primers. Lane 1: 100 bp DNA ladder; Lane 2: positive control for *S.* Typhimorium; Lanes 3, 4, 5, 6: positive samples for *S*. Typhimorium; Lane 7: negative control.

Using culture method and based on the morphology of *Salmonella* colonies on selective and non-selective media, 14 samples were positive (14%) and confirmed by PCR (a 284-bp fragment of the *invA* gene). From 14 isolates, 6 were identified as *S.* Enteritidis (a 185-bp fragment of the *prot6e* gene) (Fig. 1) and 5 as *S*. Typhimurium (a 559-bp fragment of *fliC* gene) using multiplex PCR assay (Fig. 2).

The remaining 3 isolates were not approved as *S.* Enteritidis or *S*. typhimurium.

## DISCUSSION

Estimation of the contamination rate of food with microorganisms is the first step in food poisoning control strategies. The percentage of *Salmonella* genus isolation obtained in this study was 14% using culture method and multiplex PCR. Jamshidi et al. (21) isolated *Salmonella* from 11.66% of the samples by the same method which shows an increasing trend during 3 years in Mashhad city. The contamination rate in other studies also showed, 124 (33%) in fresh chicken and beef meat samples in retail outlets of Tehran (22), 25% (60/241) in whole raw chicken samples in England (23) and also 3% in poultry carcasses in the USA (24).

The difference between the results obtained in this study and those reported by others may depend on factors like good manufacturing practices and HACCP application during the slaughter process, the process step selected for sampling, season of slaughter and the technique employed for sampling and culturing (26). In this study, samples were collected from chiller step of the poultry plant. In a study conducted in 2002 in Poland, the lowest *Salmonella* spp. contamination rate (6%) of slaughtered birds was found after stunning, and the highest contamination rate was found before chilling (52%) (26).

*S*. Enteritidis and *S*. Typhimurium are the main serovars implemented in controlling risk factors of salmonellosis because they are transmissible from parent to the chickens leaving the hatchery (27). Results showed that *S.* Entritidis was detected in 43% (6/14) and *S*. Typhimorium in 36% (5/14) of the samples and the remaining three isolates (21%) were probably belonged to other serovars which were not evaluated in the current study. Ulloa et al. (28) reported the contamination rate of *S*. Entritidis in poultry carcasses as 1.8% (25). Alexandre et al. (29) reported an isolation rate of 8.3% and 12.9% in 1154 chicken meat and chicken portion samples, respectively, with the most prevalent serotype being *S.* Entritidis. Jamshidi et al. (17) reported the contamination rate of *S*. Typhimorium as 8.3% and 6.1% in poultry carcasses in Mashhad. *S*. Entritidis was also the dominant serological type in a study conducted by Mikołajczyk and Radkowski (24). De Freitas et al. (2010) identified *S*. Entritidis in 1.37% of the poultries in Brazil by mPCR (30).

Today, for the detection and identification of *Salmonella* spp, molecular methods have enough accuracy and sensitivity especially for large number of samples (31). It was also reported that the use of two techniques could significantly increases the number of identified isolates (30) since PCR cannot distinguish between dead and living cells and it should be applied in parallel with the microbiological tests (30). In the current study both detection methods were used; the traditional technique (enrichment broth, selective media, biochemical tests) in combination with molecular technique.

In another study, *Salmonella* was recovered from 32 (16%) of the samples using traditional culture methods and in 38 (19%) of the samples by PCR method, when culture and PCR results were combined, the pathogen detection increased to 45 out of the 198 samples (23%) (13).

In conclusion, results of study showed chicken carcasses are potential vehicles of *Salmonella* spp. infection for humans. The poultry industry has to focus on implementing control measures in order to reduce the spreading of the pathogen in the production processes.
